# A core outcome set for studies of gestational diabetes mellitus prevention and treatment

**DOI:** 10.1007/s00125-020-05123-6

**Published:** 2020-03-20

**Authors:** Aoife M. Egan, Delia Bogdanet, Tomás P. Griffin, Oratile Kgosidialwa, Mila Cervar-Zivkovic, Eugene Dempsey, John Allotey, Fernanda Alvarado, Cheril Clarson, Shamil D. Cooray, Harold W. de Valk, Sander Galjaard, Mary R. Loeken, Michael J. A. Maresh, Angela Napoli, Paula M. O’Shea, Ewa Wender-Ozegowska, Mireille N. M. van Poppel, Shakila Thangaratinam, Caroline Crowther, Linda M. Biesty, Declan Devane, Fidelma P. Dunne, the INSPIRED research group

**Affiliations:** 1 Division of Endocrinology, Mayo Clinic, 200 1st Street SW, Rochester, MN 55905 USA; 2 School of Medicine, National University of Ireland Galway, Galway, Ireland; 3 Department of Endocrinology, St Vincent’s University Hospital, Dublin, Ireland; 4 Division of Obstetrics, Medizinische Universitat Graz, Graz, Austria; 5 INFANT Centre and Department of Paediatrics & Child Health, University College Cork, Cork, Ireland; 6 Barts Research Centre for Women’s Health (BARC), Women’s Health Research Unit, Blizard Institute, Barts and the London School of Medicine and Dentistry, Queen Mary University of London, London, UK; 7 Mother Infant Research Institute, Tufts Medical Center, Boston, MA USA; 8 Department of Pediatrics, University of Western Ontario, London, ON Canada; 9 Lawson Health Research Institute, London, ON Canada; 10 Diabetes and Vascular Medicine Unit, Monash Health, Melbourne, VIC Australia; 11 Monash Centre for Health Research and Implementation, Monash University, Melbourne, VIC Australia; 12 Department of Internal Medicine, University Medical Centre Utrecht, Utrecht, the Netherlands; 13 Department of Obstetrics and Gynaecology, Division of Obstetrics and Prenatal Medicine, Erasmus MC, University Medical Centre Rotterdam, ‘s-Gravendijkwal 230, 3015 CE Rotterdam, the Netherlands; 14 Section of Islet Cell and Regenerative Biology, Joslin Diabetes Center, Boston, MA USA; 15 Department of Medicine, Harvard Medical School, Boston, MA USA; 16 Department of Obstetrics, St Mary’s Hospital, Manchester University Hospitals NHS Foundation Trust, Manchester Academic Health Science Centre, Manchester, UK; 17 Department of Clinical and Molecular Medicine, Sant’Andrea University Hospital, Sapienza, University of Rome, Rome, Italy; 18 Department of Reproduction, Poznan University of Medical Sciences, Poznan, Poland; 19 Institute of Sport Science, University of Graz, Graz, Austria; 20 Institute of Metabolism and Systems Research, University of Birmingham, Birmingham, UK; 21 Liggins Institute, The University of Auckland, Auckland, New Zealand; 22 School of Nursing and Midwifery, National University of Ireland Galway, Galway, Ireland; 23 HRB-Trials Methodology Research Network, College of Medicine, Nursing and Health Sciences, National University of Ireland Galway, Galway, Ireland

**Keywords:** Clinical diabetes, Core outcome set, Insulin therapy, Pregnancy, Systematic review

## Abstract

**Aims/hypothesis:**

The aim of this systematic review was to develop core outcome sets (COSs) for trials evaluating interventions for the prevention or treatment of gestational diabetes mellitus (GDM).

**Methods:**

We identified previously reported outcomes through a systematic review of the literature. These outcomes were presented to key stakeholders (including patient representatives, researchers and clinicians) for prioritisation using a three-round, e-Delphi study. A priori consensus criteria informed which outcomes were brought forward for discussion at a face-to-face consensus meeting where the COS was finalised.

**Results:**

Our review identified 74 GDM prevention and 116 GDM treatment outcomes, which were presented to stakeholders in round 1 of the e-Delphi study. Round 1 was completed by 173 stakeholders, 70% (121/173) of whom went on to complete round 2; 84% (102/121) of round 2 responders completed round 3. Twenty-two GDM prevention outcomes and 30 GDM treatment outcomes were discussed at the consensus meeting. Owing to significant overlap between included prevention and treatment outcomes, consensus meeting stakeholders agreed to develop a single prevention/treatment COS. Fourteen outcomes were included in the final COS. These consisted of six maternal outcomes (GDM diagnosis, adherence to the intervention, hypertensive disorders of pregnancy, requirement and type of pharmacological therapy for hyperglycaemia, gestational weight gain and mode of birth) and eight neonatal outcomes (birthweight, large for gestational age, small for gestational age, gestational age at birth, preterm birth, neonatal hypoglycaemia, neonatal death and stillbirth).

**Conclusions/interpretation:**

This COS will enable future GDM prevention and treatment trials to measure similar outcomes that matter to stakeholders and facilitate comparison and combination of these studies.

**Trial registration:**

This study was registered prospectively with the Core Outcome Measures in Effectiveness Trials (COMET) database: http://www.comet-initiative.org/studies/details/686/

**Electronic supplementary material:**

The online version of this article (10.1007/s00125-020-05123-6) contains peer-reviewed but unedited supplementary material, which is available to authorised users.

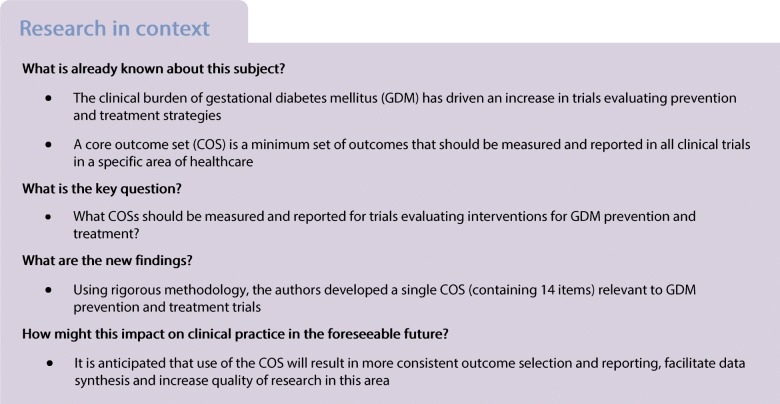

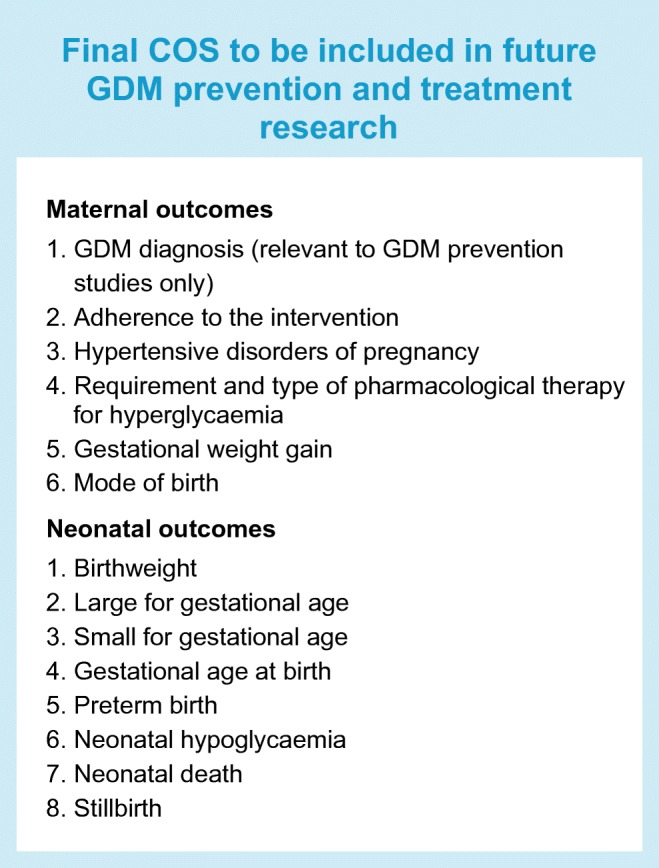





## Introduction

Gestational diabetes mellitus (GDM) is diabetes with onset or first recognition during pregnancy that was clearly not overt diabetes prior to gestation [1]. It affects 18.4 million pregnancies worldwide annually [2]. GDM is associated with an increased risk of adverse pregnancy outcomes including pre-eclampsia and Caesarean delivery for the mother and neonatal hypoglycaemia, large for gestational age and birth trauma for the infant [3–7]. These offspring are at increased risk of diabetes and obesity [8, 9] during childhood and adulthood and the mothers have a significantly elevated risk of type 2 diabetes [10, 11].

Over the past two decades, the burden of GDM has driven an increase in randomised trials of interventions for the prevention and treatment of GDM [12, 13]. However, heterogeneity in outcomes reported in these trials makes combining and comparing results difficult [14]. As a result, evidence synthesis and meta-analyses become less efficient and the reliability of evidence to guide healthcare decisions is limited [15]. One approach to address this lack of uniformity is to develop a core outcome set (COS) or an agreed set of outcomes [16]. A COS represents a minimum set of outcomes that are expected to be measured and reported in all trials in specific areas of healthcare; however, researchers may report additional outcomes at their discretion. Typically, COSs are also suitable for use in relevant clinical audits and observational studies [17]. The Core Outcome Measures in Effectiveness Trials (COMET) initiative brings together current thinking and provides methodological guidance on this subject [17, 18]. In the field of women’s health, over 50 journals endorse the Core Outcomes in Women’s Health (CROWN) initiative, which promotes COS development and effective dissemination of related manuscripts [19].

The aim of this study was to develop COSs for studies evaluating the effectiveness of interventions for the prevention or treatment of GDM.

## Methods

This study was registered prospectively with the COMET database (http://www.comet-initiative.org/studies/details/686/) and a detailed protocol is published [20]. The study design was guided by the COMET initiative and COS-STAD recommendations [18, 21]. Ethical approval for the study was obtained from the Galway University Hospitals ethics committee.

**Fig. 1:**
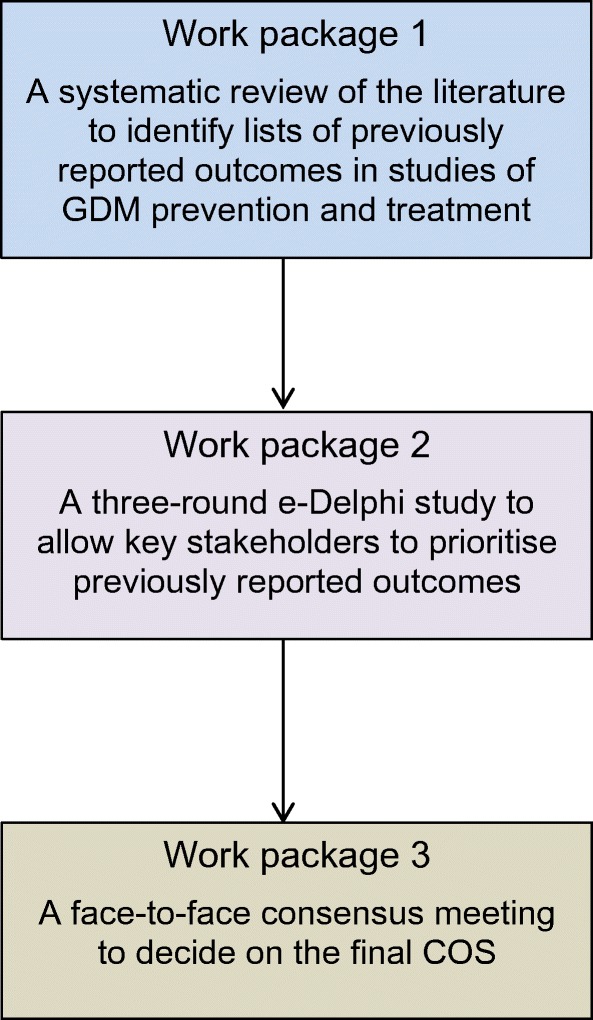
Summary of the study work packages

The study was conducted within three work packages (Fig. 1): (1) a systematic review of the literature to identify previously reported outcomes; (2) a three-round, web-based, e-Delphi survey with key stakeholders to prioritise outcomes; and (3) a consensus meeting to finalise the COS.

Two separate procedures were originally planned and conducted: one for GDM prevention and one for GDM treatment. However, the results from these separate consensus procedures were very similar and stakeholders at the consensus meeting decided to produce a single COS for all studies of GDM prevention or treatment.

### Systematic review

The search strategy is outlined in the study protocol. In brief, the following databases were searched for relevant studies: MEDLINE; Embase; Web of Science; Cochrane Library; and the Cumulative Index to Nursing and Allied Health Literature (CINAHL). We included randomised trials and systematic reviews of randomised trials (with and without meta-analyses) comparing the effectiveness of pharmacological and non-pharmacological interventional strategies for both prevention and treatment of GDM. Given the large number of previously published studies, a pragmatic approach was taken and the systematic review was performed in stages until outcome saturation was reached. In this regard, the initial search included time limits of publication between January 2015 and February 2019.

Two reviewers (A.M. Egan and D. Bogdanet) assessed the titles and abstracts of identified studies independently. Full texts of studies meeting the inclusion criteria (or where this was uncertain) were retrieved and consensus was reached on inclusion status. Studies were divided into those for prevention and those for treatment of GDM. Outcomes were extracted and grouped under three major domains following review by the study advisory group (see study protocol) [20]: maternal outcomes; neonatal outcomes; and other outcomes.

### e-Delphi study

We conducted a three-round, e-Delphi study using SurveyMethods software (www.surveymethods.com, accessed 16 April 2019). Participants were recruited from the following groups: (1) women representatives (pregnant women at risk of GDM, with GDM or women with a history of GDM); (2) healthcare professionals (professionals who care for women with GDM); and researchers (researchers and policy makers with an active interest in GDM).

We sent an e-mail explaining the study and inviting participation through an electronic link to the list managers of the following organisations: International Association of the Diabetes and Pregnancy Study Groups (IADPSG); Diabetes Ireland (DI); Irish Endocrine Society (IES); ADA; EASD; the Irish Midwifery e-Group; and the Diabetic Pregnancy Study Group (DPSG) of the EASD. We also circulated information about the study on social media and to women with diabetes attending antenatal clinics at University Hospital Galway, Galway, Ireland. Potential participants were invited to forward the invitation to others whom they regarded as having the required expertise. An e-mail reminder was sent to anyone who did not respond after 10 days and again after 14 days.

The round 1 survey included a further explanation of the study with a consent process and followed with a short questionnaire that requested participant demographic data including the stakeholder group that best represented their profile. It also presented the outcomes identified in the review and participants were asked to rate each outcome for GDM prevention and treatment separately on a nine-point Likert-scale with higher values representing increased importance for inclusion in the COS. There was an ‘unable to score’ option that could be selected for each outcome. All outcomes were accompanied by a plain English explanation. Participants were invited to submit up to two additional outcomes for GDM prevention and two additional outcomes for GDM treatment that they considered important or relevant for inclusion in the COS [18]. The results from round 1 were summarised using descriptive statistics and all outcomes were carried forward to round 2 including additional outcomes that were suggested by more than one participant.

Participants who completed round 1 were invited to participate in round 2. In this second round, participants were provided with their scores from round 1 and with the distribution of scores for each outcome per stakeholder group. They were asked to re-score the outcomes after considering the information provided from round 1. Outcomes were classified as ‘consensus in’ (≥70% participants giving scores of 7–9 and <15% scoring 1–3), ‘consensus out’ (≤50% participants scoring 7–9 in each stakeholder group) or ‘no consensus’ (anything else). All outcomes scored as ‘consensus in’ were carried forward to round 3. All participants who completed rounds 1 and 2 were invited to participate in round 3. Again, participants were provided with their scores from round 2 and the distribution of scores per stakeholder group. Participants were asked to re-score the outcomes.

### Consensus meeting

A face-to-face consensus meeting took place on 5 September 2019 in Graz, Austria. The aim was to agree on the final outcomes to be included in the COS. The meeting was moderated by an experienced chairperson (DD) who did not vote at the meeting. Outcomes classified as ‘consensus in’ or ‘no consensus’ in the e-Delphi round 3 were presented at the meeting along with the responses per stakeholder group. An open discussion took place on each outcome. There was opportunity to combine or modify individual outcomes and participants were encouraged to consider whether each outcome was applicable to GDM treatment or GDM prevention or both. Participants were then asked to vote on each outcome as ‘yes’ or ‘no’ for inclusion in the COS. Participants used Poll Everywhere (www.polleverywhere.com, accessed 16 October 2019) to vote anonymously. An outcome was included in the final COS when ≥70% participants voted ‘yes’.

## Results

### Systematic review

**Fig. 2:**
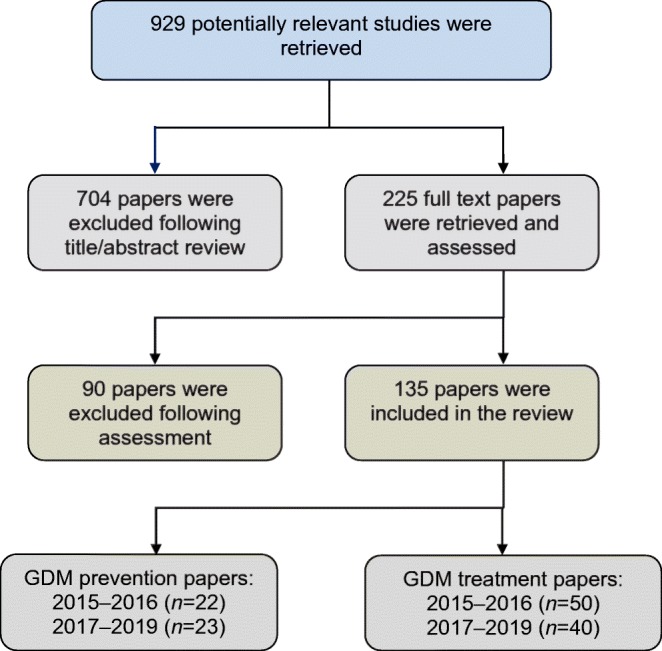
Selection of studies for systematic review

Electronic supplementary material (ESM) Fig. 1 includes the PRISMA flow diagram which depicts the flow of information through the different phases of the systematic review. A total of 929 potentially relevant studies were retrieved. Following review of the title and abstract, 225 full text papers were retrieved and assessed. Ninety papers were excluded following assessment and 135 papers were included in the review. Of the 135 papers identified, 45 related to GDM prevention and 90 related to GDM treatment (Fig. 2). ESM Table 1 lists all included studies. Outcomes from 2017–2019 studies were indexed initially followed by outcomes from 2016 and 2015. During extraction of 2015 outcomes, saturation was reached, with no new additional outcomes identified during this time period. Extracted outcomes were reviewed by the study advisory group to ensure removal of duplicate outcomes, combine similar outcomes and clarify outcome terminology. Following this, 74 GDM prevention outcomes and 116 GDM treatment outcomes were listed for inclusion in round 1 of the e-Delphi Study (ESM Table 2).

### e-Delphi study

Round one was completed by 173 stakeholders (*n* = 132, 76% female sex; *n* = 39, 23% male sex; *n* = 2, 1.0% did not disclose sex). All participants gave informed consent prior to participating. There was international distribution of participants with 27 countries and six continents represented (ESM Table 3). A total of 69 (40%) respondents were from Ireland, 20 (12%) were from Canada and 16 (9%) from the USA. Stakeholders represented three broad categories: patient representatives (*n* = 23, 13%); healthcare professionals (*n* = 116, 67%); and researchers (*n* = 34, 19%). Within the group who self-identified as ‘healthcare professionals’, there was representation from the following disciplines: anaesthesiology; midwifery; specialist midwifery; dietetics; endocrinology; general practice; neonatology; specialist nursing; obstetric medicine; obstetrics; paediatrics; pharmacy; and physiotherapy.

The round 2 survey again presented the lists of GDM prevention outcomes and GDM treatment outcomes. One additional outcome (breastfeeding) was included in the list of GDM prevention outcomes as it was suggested by more than one participant in round 1. Round 2 was completed by 70% (121/173) of those who had completed the first survey as follows: patient representatives, *n* = 19 (16%); healthcare professionals, *n* = 70 (58%); and researchers, *n* = 32 (26%).

A total of 22 GDM prevention outcomes and 30 GDM treatment outcomes were classified as ‘consensus in’ and were carried forward to round 3 (ESM Table 4). Round 3 was completed by 84% (102/121) of those who had completed round 2 as follows: patient representatives, *n* = 16 (16%); healthcare professionals, *n* = 56 (55%); and researchers, *n* = 30 (29%). Following analysis of round 3, all outcomes were classified as ‘consensus in’ or ‘no consensus’ and therefore all 22 GDM prevention and 30 GDM treatment outcomes were carried forward to the face-to-face consensus meeting.

### Consensus meeting

The panel consisted of 23 participants, representing a variety of countries, who had volunteered to take part in the e-Delphi study or who had been sampled purposefully by the study advisory group. The participants included representatives from each of the three stakeholder groups: patient representatives (*n* = 6); healthcare professionals (*n* = 10); and researchers (*n* = 7). In addition to the non-voting chairperson, there were two administrators responsible for recording the discussion and poll results. ESM Table 4 outlines the results of the voting at the consensus meeting for each outcome. Based on the views of the group, the treatment outcome listed as ‘requirement for pharmacological therapy for hyperglycaemia’ was rephrased to ‘requirement and type of pharmacological therapy for hyperglycaemia’ and the treatment outcome listed as ‘perinatal mortality’ was changed to ‘neonatal death’. Following the voting process, 11 outcomes were included in the GDM prevention COS and 13 were included in the GDM treatment COS (ESM Table 4). All eight chosen outcomes from the neonatal domain were identical between the prevention and treatment COSs. The GDM treatment COS contained two additional outcomes that were not included in the GDM prevention COS. These were ‘adherence to the intervention’ and ‘mode of birth’. Following a further discussion and vote, these outcomes were included in the GDM prevention COS. Finally, the prevention outcome ‘GDM diagnosis’ was (appropriately) not included in the GDM treatment COS. Given the fact that there was agreement on all other outcomes between the two COSs, it was decided that a single COS for the prevention and/or treatment of GDM would be ideal and would likely increase uptake of the COS.

Text box 1 outlines the final COS, which includes six maternal and eight neonatal outcomes. The outcome ‘GDM prevention’ is highlighted as relevant to GDM prevention studies only.

## Discussion

In this study, a global group of key stakeholders agreed on 14 outcomes to form a COS essential for future trials of GDM prevention or treatment (Table 1). These outcomes are grouped under two domains including six maternal and eight neonatal outcomes. Although the COS was developed with a specific focus on randomised trials, it should be useful for non-randomised studies and audit in this field [17]. It is anticipated that this COS will improve consistency in outcome reporting, facilitate data synthesis and increase the quality of research relevant to GDM prevention and treatment. The formation of this COS responds to previous calls for the development of a COS in this area to reduce research waste and improve health outcomes for women with GDM [22].

**Table 1:** Final COS to be included in future GDM prevention and treatment research

Domain	Outcome
Maternal outcomes	1. GDM diagnosis^a^
	2. Adherence to the intervention
	3. Hypertensive disorders of pregnancy
	4. Requirement and type of pharmacological therapy for hyperglycaemia
	5. Gestational weight gain
	6. Mode of birth
Neonatal outcomes	1. Birthweight
	2. Large for gestational age
	3. Small for gestational age
	4. Gestational age at birth
	5. Preterm birth
	6. Neonatal hypoglycaemia 7. Neonatal death
	8. Stillbirth

^a^Relevant to GDM prevention studies only

The COMET handbook [18] and the Core Outcome Set–Standards for Development (COS-STAD) [21] were used to guide the development of this study and a detailed protocol was published. The three-step approach involving a systematic review, e-Delphi survey and consensus meeting has been used widely in COS development [16, 23, 24]. Given the extensive body of published literature in the area of GDM prevention and treatment, the study team used sequential searching with relatively narrow time limits until outcome saturation was reached. It was believed that an exhaustive search of previously reported trials with no time limit would require extensive resources and would likely be of low yield. This pragmatic method yielded a comprehensive list with a total of 190 outcomes available for rating in round 1 of the e-Delphi study. In this next step of the study, a large and international group of stakeholders prioritised the identified outcomes. This method allows participants to have an equal voice in rating and to suggest additional outcomes for consideration in the next round of the e-Delphi. Participants were limited to submitting two additional GDM prevention and two additional GDM treatment outcomes; additional outcomes were carried forward only if suggested by more than one stakeholder. Based on prior experience, additional outcomes are very unlikely to be included in the final COS if suggested by just one person and we wished to avoid survey fatigue by extending an already long survey [16, 24]. The consensus meeting brought together a diverse group including women representatives, researchers and clinicians of varying backgrounds. A broad range of viewpoints was heard and the chairperson facilitated this. Special attention was taken to ensure that women representatives were given the opportunity to take part actively and plain language explanations were provided for each outcome under discussion. Two healthcare professionals were charged with providing further explanations on outcomes particularly to women representatives. This seemed to enhance the participation of the women representatives. The use of an anonymous voting system prevented participants feeling pressurised into voting a specific way following the group discussion. During the meeting, the women representatives shared many personal experiences in order to highlight the real-life impact of a specific outcome, and this was valued within the group.

The systematic review was limited to English language publications. This may have introduced a selection bias, although given the large number of included studies from a variety of centres internationally the likelihood of missing important outcomes is low. We did not introduce a qualitative aspect to the first phase of the study, such as semi-structured interviews. It may be argued that this could minimise patient involvement and the number of patient-centred outcomes included in the e-Delphi study. However, women representatives were included at every stage of the study and were active participants in the core study group, the study advisory group, the e-Delphi process (with the opportunity to add additional outcomes) and the consensus meeting. In addition, outcomes were only excluded during the e-Delphi stage if ≤50% participants scored them as 7–9 in each of the stakeholder groups. This resulted in more outcomes being brought forward to the consensus meeting but gave stakeholder representatives the opportunity to explain their rationale for marking an outcome highly. We adapted a snowball sampling approach for the e-Delphi study. This allowed participants to recruit additional participants but meant that we did not know how many potential participants actually responded to the survey. However, we did exceed our specified goal of at least 20 respondents from each stakeholder group in round 1 [20] and the retention rates of 70% between rounds 1 and 2 and 84% between rounds 2 and 3 compare well to prior COS studies [15, 16]. The greatest non-response rate to rounds 2 and 3 were among healthcare professionals. Interestingly, this group had formed the majority of participants in round 1. The implications of this with respect to the final COS is unclear, although we are reassured that this group still had significant representation at each point in the study. While study participants had a broad range of backgrounds and countries of residence, developing countries were not well represented. This may limit generalisability of the study to these areas of the world and future work should explore this issue in more detail. Finally, the scope of this study was to identify ‘what’ and not ‘how’ outcomes should be collected. There is a published repository of acceptable definitions relating to diabetes in pregnancy outcomes that may be referenced by researchers in order to define ‘how’ outcomes can be collected [25].

The issue of presenting one COS applicable to both GDM prevention and treatment studies was discussed in detail at the face-to-face meeting and a final decision was based on an electronic vote that was unanimous in favour of combining. This approach was recently taken by COSGROVE study researchers who developed a COS for prevention and treatment of fetal growth restriction [15]. The next important step will be to ensure effective dissemination and uptake of the COS and it was the group consensus that having one COS, rather than two, would facilitate this process.

In summary, this is the first study to define a COS in the area of GDM prevention and treatment. It is anticipated that these outcomes, considered essential by key stakeholders, are collected in future trials and will have a positive impact on the ability to compare and combine studies. This will allow better assessment of the effect of a specific intervention, particularly in relation to rare but important outcomes (such as stillbirth and neonatal death), that individual studies may not be adequately powered to assess. The authors now call on funders, researchers and journals to incorporate this COS into relevant studies with the aim of improving research in the field of GDM and ultimately outcomes for women with GDM and their offspring.

## Electronic supplementary material

ESM Table 1 (XLS 91 kb)

ESM (PDF 281 kb)

## Data Availability

Data are available on request from the authors.

## Abbreviations

COMET: Core Outcome Measures in Effectiveness Trials
COS: Core outcome set
GDM: Gestational diabetes mellitus
