# Supplementary material for: A core outcome set for studies of gestational diabetes mellitus prevention and treatment
Source: Diabetologia. 2020 Mar 20;63(6):1120–7. doi: 10.1007/s00125-020-05123-6 (PMC7228989; doi:10.1007/s00125-020-05123-6)
Supplement: Supplementary file 2 — (PDF 281 kb) [file 125_2020_5123_MOESM2_ESM.pdf]

ESM Table 2: Outcomes included in e-Delphi Round 1 and number and percentage of respondents giving each outcome a "high" score of 7-9.

	Group 1: Patient Representatives<br><br>Scoring each outcome 7-9			Group 2: Health Care Professionals<br><br>Scoring each outcome 7-9			Group 3: Researchers<br><br>Scoring each outcome 7-9	
**Prevention Outcome**	**n = 23**	**%**		**n = 116**	**%**		**n = 34**	**%**
**1. Maternal Outcomes**								
GDM Diagnosis	21	91%		108	93%		32	94%
Gestational Weight Gain	17	74%		93	80%		26	76%
Increase in BMI during pregnancy	13	57%		72	62%		21	62%
Body Composition	12	52%		41	35%		14	41%
Skin Fold thickness	9	39%		30	26%		12	35%
Waist Circumference	12	52%		44	38%		15	44%
Postpartum weight retention	14	61%		79	68%		26	76%
Requirement for insulin therapy	20	87%		105	91%		32	94%
Blood pressure	17	74%		88	76%		28	82%
Hypertensive disorders of pregnancy	20	87%		90	78%		30	88%

Maternal hospitalisation	12	52%		71	61%		24	71%
Placental abruption	17	74%		59	51%		23	68%
Dietary intake	18	78%		91	78%		24	71%
Physical activity	16	70%		90	78%		24	71%
Self-rated health	13	57%		63	54%		21	62%
Quality of life	16	70%		74	64%		23	68%
Self-rated diet	12	52%		54	47%		15	44%
Induction of labour	11	48%		63	54%		20	59%
Mode of birth	15	65%		81	70%		28	82%
Termination of pregnancy	7	30%		56	48%		16	47%
Miscarriage	14	61%		66	57%		18	53%
Maternal accident	12	52%		31	27%		10	29%
Maternal sepsis	14	61%		57	49%		19	56%
Hospitalisation during the pregnancy	13	57%		62	53%		21	62%
Admission to the HDU	14	61%		67	58%		23	68%
Intrapartum haemorrhage	15	65%		52	45%		19	56%
Postpartum haemorrhage	15	65%		54	47%		23	68%
Perineal trauma	10	43%		59	51%		23	68%
Fasting blood glucose	18	78%		103	89%		30	88%
Postprandial glucose	18	78%		97	84%		27	79%
Insulin	17	74%		58	50%		22	65%
Hba1c	18	78%		91	78%		28	82%
C peptide	14	61%		57	49%		15	44%
Ferritin	12	52%		33	28%		14	41%
Maternal lipid profile	12	52%		59	51%		14	41%
Haemoglobin	12	52%		43	37%		11	32%
High sensitivity CRP	13	57%		30	26%		9	26%
HOMA-IR	15	65%		59	51%		20	59%

IL-6	11	48%		24	21%		6	18%
Leptin	11	48%		27	23%		4	12%
Cortisol	11	48%		30	26%		5	15%
Non-esterified fatty acids	9	39%		27	23%		9	26%
Ratio of plasminogen activator inhibitor 1 to 2	9	39%		23	20%		13	38%
Vitamin D level	13	57%		45	39%		6	18%
**2. Neonatal Outcomes**								
Preterm birth	17	74%		83	72%		31	91%
Gestational week at birth	17	74%		95	82%		32	94%
Neonatal death	18	78%		91	78%		31	91%
Stillbirth	18	78%		90	78%		31	91%
Small for gestational age	16	70%		87	75%		30	88%
Large for gestational age	18	78%		101	87%		33	97%
Macrosomia	16	70%		94	81%		29	85%
Birthweight	19	83%		99	85%		32	94%
Skinfold thickness	14	61%		44	38%		17	50%
Baby anthropometry	17	74%		74	64%		25	74%
% body fat neonate	13	57%		50	43%		19	56%
APGAR	15	65%		77	66%		27	79%
Pondoral index	10	43%		55	47%		20	59%
Respiratory distress	16	70%		75	65%		26	76%
Hyperbilirubinemia	13	57%		73	63%		25	74%
Congenital malformation	14	61%		82	71%		26	76%
Brachial plexus injury	12	52%		89	77%		29	85%
Bone fracture	13	57%		76	66%		27	79%
Shoulder dystocia	11	48%		94	81%		28	82%
Neonatal sepsis	15	65%		67	58%		21	62%
Retinopathy of	14	61%		54	47%		18	53%

prematurity								
Neonatal hypoglycaemia	18	78%		99	85%		29	85%
Neonatal internal haemorrhage	16	70%		60	52%		18	53%
Need for mechanical ventilation	16	70%		71	61%		20	59%
Necrotising enterocolitis	15	65%		54	47%		19	56%
Admission to neonatal ICU	16	70%		91	78%		27	79%
Number of days in special baby care unit	13	57%		83	72%		27	79%
Number of days in hospital	13	57%		79	68%		25	74%
Discharge home on oxygen	13	57%		52	45%		16	47%
**3. Other Outcomes**								
Health cost analysis	14	61%		71	61%		23	68%

	Group 1: Patient Representatives<br><br>Scoring each outcome 7-9			Group 2: Health Care Professionals<br><br>Scoring each outcome 7-9			Group 3: Researchers<br><br>Scoring each outcome 7-9	
**Treatment Outcome**	**n = 23**	**%**		**n = 116**	**%**		**n = 34**	**%**
**1. Maternal Outcomes**								
Quality of life score	15	65%		68	59%		24	71%
Self-care behaviour	17	74%		67	58%		19	56%
Satisfaction with treatment	15	65%		71	61%		22	65%
Empowerment	16	70%		63	54%		16	47%
Self-efficacy	14	61%		60	52%		18	53%
Health related quality of life	14	61%		62	53%		22	65%
Compliance with self monitoring	16	70%		85	73%		23	68%
Depression	16	70%		68	59%		28	82%
Stress	18	78%		66	57%		21	62%
Post natal depression	15	65%		69	59%		26	76%

Adherence to the intervention	15	65%		85	73%		28	82%
Behaviour change associated with the intervention	16	70%		77	66%		23	68%
Anxiety	16	70%		59	51%		21	62%
Cost of treatment	13	57%		75	65%		25	74%
Acceptability of treatment	14	61%		81	70%		29	85%
Vitamin D level	12	52%		53	46%		13	38%
Glucose fasting	18	78%		90	78%		28	82%
1 hour glucose tolerance test result	19	83%		80	69%		24	71%
2 hour glucose tolerance test result	19	83%		91	78%		28	82%
Post-prandial glucose level	18	78%		83	72%		22	65%
Average glucose level	17	74%		63	54%		20	59%
% glucose measurements out of range	17	74%		75	65%		17	50%
Time to control of glucose level	17	74%		71	61%		17	50%
Insulin level	17	74%		41	35%		16	47%
HbA1c	18	78%		82	71%		23	68%
HOMA-IR	15	65%		49	42%		15	44%
Very low density lipoprotein cholesterol	11	48%		44	38%		14	41%
Free fatty acids	12	52%		39	34%		14	41%
Lipid profile	12	52%		54	47%		17	50%

High sensitivity CRP	13	57%		34	29%		14	41%
QUICKI	14	61%		35	30%		12	35%
C peptide	13	57%		36	31%		10	29%
Carbohydrate intake per day	17	74%		72	62%		22	65%
Protein intake per day	16	70%		64	55%		19	56%
Fat intake per day	13	57%		62	53%		21	62%
Blood pressure	19	83%		83	72%		26	76%
HELLP Syndrome	14	61%		70	60%		24	71%
Hypertensive disorders of pregnancy	16	70%		78	67%		29	85%
Number of hospitalisations	12	52%		75	65%		25	74%
Polyhydramnios	16	70%		82	71%		22	65%
Placental abruption	16	70%		68	59%		23	68%
Requirement for insulin	18	78%		92	79%		29	85%
Gestational age at insulin therapy	15	65%		89	77%		26	76%
Total daily insulin dose	19	83%		81	70%		25	74%
Requirement for metformin	17	74%		81	70%		21	62%
Requirement for pharmacological therapy for hyperglycaemia	18	78%		89	77%		29	85%
Hypoglycaemia	15	65%		78	67%		27	79%
Treatment failure	16	70%		80	69%		28	82%
Gestational weight gain	17	74%		89	77%		30	88%
Change in BMI	19	83%		70	60%		21	62%
Maternal weight at time	16	70%		76	66%		22	65%

of birth								
Return to prepregnancy weight	17	74%		76	66%		24	71%
Induction of labour	14	61%		70	60%		21	62%
Prolonged labour	12	52%		52	45%		19	56%
Duration of labour	12	52%		54	47%		15	44%
Premature rupture of membranes	12	52%		54	47%		17	50%
Birth complication	17	74%		79	68%		28	82%
Mode of birth	15	65%		81	70%		29	85%
Reason for caesarean birth	15	65%		82	71%		27	79%
Perineal trauma	12	52%		71	61%		22	65%
Blood loss during birth	14	61%		48	41%		19	56%
Post partum haemorrhage	15	65%		59	51%		21	62%
Chorioamnionitis	13	57%		55	47%		17	50%
Maternal ICU admission	14	61%		74	64%		26	76%
Postpartum infection	13	57%		59	51%		22	65%
Breast feeding	14	61%		78	67%		29	85%
Maternal mortality	16	70%		82	71%		31	91%
Maternal serious morbidity	16	70%		83	72%		29	85%
Development of type 2 diabetes	19	83%		94	81%		32	94%
Post pregnancy weight	17	74%		83	72%		26	76%
**2. Neonatal Outcomes**								
Fetal growth restriction	14	61%		82	71%		25	74%
Macrosomia	16	70%		87	75%		26	76%

Birthweight	16	70%		91	78%		31	91%
Large for gestational age	15	65%		90	78%		31	91%
Small for gestational age	13	57%		84	72%		30	88%
Gestational age at birth	17	74%		92	79%		29	85%
Preterm birth	16	70%		83	72%		22	65%
Neonatal arm circumference	11	48%		48	41%		14	41%
Birth length	12	52%		57	49%		21	62%
Neonatal chest circumference	11	48%		40	34%		17	50%
Neonatal head circumference	12	52%		52	45%		20	59%
APGAR	15	65%		72	62%		28	82%
Congenital malformation	15	65%		76	66%		25	74%
Pondoral index	14	61%		54	47%		21	62%
Shoulder dystocia	13	57%		86	74%		26	76%
Bone fracture	13	57%		70	60%		25	74%
Brachial plexus injury	13	57%		75	65%		27	79%
Neonatal mortality	15	65%		86	74%		28	82%
Birth trauma	15	65%		82	71%		27	79%
Neonatal hypoglycaemia	16	70%		93	80%		28	82%
Need for IV glucose	18	78%		83	72%		25	74%
Neonatal glucose level	18	78%		76	66%		24	71%
Neonatal sepsis	14	61%		63	54%		23	68%
Neonatal respiratory distress syndrome	14	61%		74	64%		24	71%
Transient tachypnoea of the newborn	13	57%		65	56%		21	62%
Bronchopulmonary	13	57%		51	44%		17	50%

dysplasia								
Neonatal internal haemorrhage	13	57%		53	46%		17	50%
Necrotising enterocolitis	12	52%		48	41%		15	44%
Hyperbilirubinemia	11	48%		66	57%		23	68%
Need for phototherapy	11	48%		60	52%		20	59%
Neonatal intensive care unit admission	15	65%		80	69%		25	74%
Infant sex	9	39%		50	43%		21	62%
Neonatal hypocalcaemia	13	57%		45	39%		14	41%
Umbilical cord PH	11	48%		56	48%		14	41%
Miscarriage	14	61%		69	59%		23	68%
Neonatal mortality	15	65%		85	73%		29	85%
Neonatal hospitalisation	14	61%		78	67%		27	79%
Livebirth	16	70%		81	70%		28	82%
Stillbirth	16	70%		82	71%		29	85%
Perinatal death	16	70%		85	73%		29	85%
Diabetes in adulthood	17	74%		81	70%		26	76%
Adiposity in adulthood	16	70%		74	64%		22	65%
Neurosensory disability in later childhood	14	61%		55	47%		18	53%
Childhood adiposity	14	61%		74	64%		23	68%
Neonatal adiposity	14	61%		70	60%		23	68%
Childhood BMI	14	61%		71	61%		21	62%

ESM Table 3: e-Delphi round 1 participants

Argentina	9	5.2%
Australia	3	1.7%
Austria	6	3.5%
Canada	20	11.6%
China	1	0.6%
Colombia	1	0.6%
Denmark	6	3.5%
UK	8	4.6%
France	2	1.2%
Germany	1	0.6%
Greece	2	1.2%
India	2	1.2%
Ireland	69	39.9%
Italy	5	2.9%
Japan	1	0.6%
Lithuania	1	0.6%
Malta	3	1.7%
Morocco	1	0.6%
The Netherlands	6	3.5%
New Zealand	3	1.7%
Poland	1	0.6%
Romania	1	0.6%
Saudia Arabia	1	0.6%
Singapore	1	0.6%
Spain	1	0.6%
Sweden	1	0.6%
USA	16	9.2%
**Total**	**173**	**100.0%**

ESM Table 4: List of GDM prevention and treatment outcomes carried forward from round 2 and their status following round three voting and discussion at the consensus meeting.

Prevention Outcomes	Consensus following Round 3 vote	Consensus following meeting
**1. Maternal Outcomes**		
GDM Diagnosis	Consensus in	Consensus in
Gestational Weight Gain	Consensus in	Consensus in
Requirement for insulin therapy	Consensus in	Consensus out
Blood pressure	No consensus	Consensus out
Hypertensive disorders of pregnancy	Consensus in	Consensus in
Physical activity	No consensus	Consensus out
Fasting blood glucose	Consensus in	Consensus out
Postprandial glucose	Consensus in	Consensus out
Hba1c	Consensus in	Consensus out
**2. Neonatal Outcomes**		
Preterm birth	Consensus in	Consensus in
Gestational week at birth[a]	Consensus in	Consensus in
Neonatal death	Consensus in	Consensus in
Stillbirth	Consensus in	Consensus in
Small for gestational age	Consensus in	Consensus in
Large for gestational age	Consensus in	Consensus in
Macrosomia	Consensus in	Consensus out
Birthweight	Consensus in	Consensus in
Congenital malformation	No consensus	Consensus out
Brachial plexus injury	No consensus	Consensus out
Shoulder dystocia	Consensus in	Consensus out
Neonatal hypoglycemia	Consensus in	Consensus in
Admission to neonatal ICU	Consensus in	Consensus out

Treatment Outcomes	Consensus following Round 3 vote	Consensus following meeting
**1. Maternal Outcomes**		
Adherence to the intervention	Consensus in	Consensus in
Glucose fasting	Consensus in	Consensus out
1 hour glucose tolerance test result	Consensus in	Consensus out
2 hour glucose tolerance test result	Consensus in	Consensus out
Hypertensive disorders of pregnancy	Consensus in	Consensus in
Requirement for insulin	Consensus in	Consensus out
Gestational age at insulin therapy	Consensus in	Consensus out
Total daily insulin dose	No consensus	Consensus out
Requirement for pharmacological therapy for hyperglycaemia[b]	Consensus in	Consensus in
Gestational weight gain	Consensus in	Consensus in
Birth complication	Consensus in	Consensus out
Mode of birth	Consensus in	Consensus in
Reason for caesarean birth	No consensus	Consensus out
Maternal mortality	Consensus in	Consensus out
Maternal serious morbidity	Consensus in	Consensus out
Development of type 2 diabetes	Consensus in	Consensus out
**2. Neonatal Outcomes**		
Macrosomia	Consensus in	Consensus out
Birthweight	Consensus in	Consensus in
Large for gestational age	Consensus in	Consensus in
Small for gestational age	Consensus in	Consensus in
Gestational age at birth	Consensus in	Consensus in
Preterm birth	Consensus in	Consensus in
Shoulder dystocia	Consensus in	Consensus out

Neonatal mortality[c]	Consensus in	Consensus in
Neonatal hypoglycaemia	Consensus in	Consensus in
Need for IV glucose	Consensus in	Consensus out
Neonatal intensive care unit admission	Consensus in	Consensus out
Livebirth	Consensus in	Consensus out
Stillbirth	Consensus in	Consensus in
Perinatal death	Consensus in	Consensus out

[a]rephrased at consensus meeting to “gestational age at birth”
[b]rephrased at consensus meeting to “requirement and type of pharmacological therapy”
[c]rephrased at consensus meeting to “neonatal death”

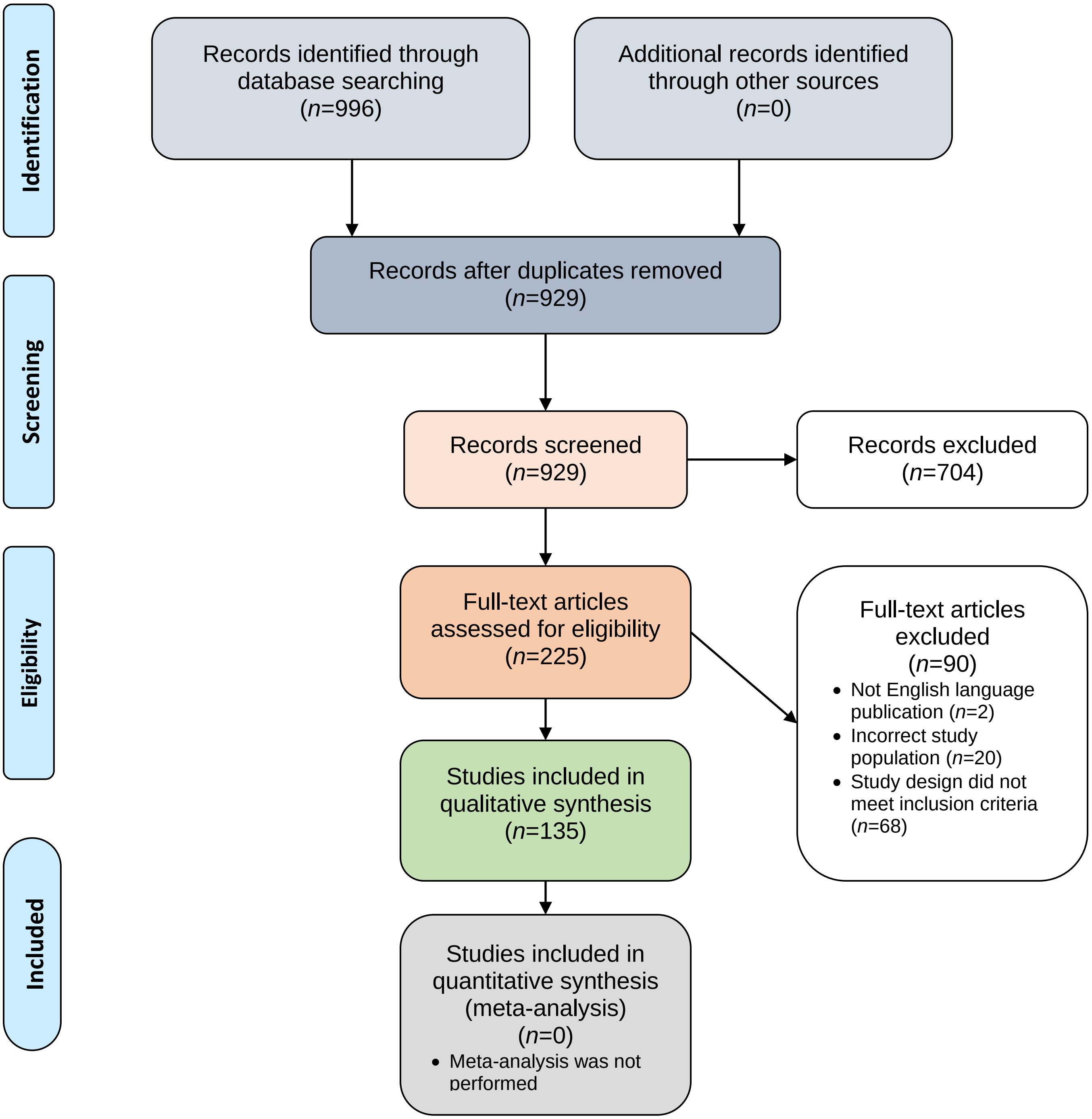
Identification
Screening
Eligibility
Included
Records identified through database searching (n=996)
Additional records identified through other sources (n=0)
Records after duplicates removed (n=929)
Records screened (n=929)
Records excluded (n=704)
Full-text articles assessed for eligibility (n=225)
Full-text articles excluded (n=90)
Not English language publication (n=2)
Incorrect study population (n=20)
Study design did not meet inclusion criteria (n=68)
Studies included in qualitative synthesis (n=135)
Studies included in quantitative synthesis (meta-analysis) (n=0)
Meta-analysis was not performed
